# Observations on Infantile Gangrene

**Published:** 1839-04

**Authors:** 


### ART. VII.

*Bemerkungen über den Brand der Kinder.* Von Dr. ADOLPH LEOPOLD RICHTER. &c.—*Berlin,* 1834. 4to. pp. 22.

*Observations on Infantile Gangrene.* By Dr. A. L. RICHTER.—*Berlin,* 1834. 4to. pp. 22.

THIS short essay was published on the occasion of celebrating the completion of the fiftieth year of public service, by the venerable military Physician-General, Dr. Von Wiebel, now four years since. It has, however, only recenly come into our hands, and we deem its contents of too much value to be kept any longer from the knowledge of our readers.

Under the term Kinderbrand, or Infantile Gangrene, are included by Dr. Richter three varieties of the same disease, or what have been described by others as three distinct diseases, occurring in infants, viz. cancer aquaticus, water-canker or noma, sphacelus of the labia, and infantile gangrene, or gangrene of the newly-born. These several affections are considered as merely varieties of one and the same disease, differing chiefly in the situation which they occupy, and the nature of the animal organs and tissues involved. The points of similarity by which he thinks their essential identity is established are as follows: the occurrence of the several varieties in the most tender age of infant life, in children who through defective nursing and nourishment, dyscrasia, and exhaustion left by preceding diseases, have been much debilitated; the simultaneous development of more than one of these forms in the same individual; the formation of a grey lead-coloured rounded spot (in the mouth and upon the parts of generation,) followed by tense swelling and hardness of the part, which is, at its circumference, of a pale red tint, passing gradually into the colour of the surrounding skin; quick transition of the grey-colour into black; rapid extension of the disease and destruction of the surrounding parts by the formation of a dry gangrenous eschar, or in a few cases of a sphacelating ulcer; and general sinking terminating in death; or, if the remedies used are successful in limiting the spread of the disease, the separation of the mortified parts by the formation of a line of demarcation and surrounding inflammation, with rapid restoration of the parts destroyed in cases where the infantile organism will bear the reaction.

Notwithstanding these points of resemblance, we are inclined to question the propriety of classing, under one name, affections apparently differing so much from each other as do those now under consideration. One important end of nosological distinction is, that we may the more readily acquire correct information respecting the various disorders which we are called upon to treat; and it would doubtless add much to the apparent simplicity of our systems, to bring together under one head, all those diseases which are in essence the same—for instance, pleuritis and peritonitis, bronchitis and muco-enteritis, &c. But we question much whether such a system would be found equally simple at the bedside of the patient, or that it would lead to any improvement in practical results. It is, however, eminently useful to bear in mind the analogies, or, if the phrase is preferred, the intimate relations of disease: the objection is to the carrying of system to such an extent as to interfere with that closeness of description to which the separate consideration of the same diseased action, manifesting itself under different circumstances of locality, whether of region, organ, or tissue, so clearly leads.

1. *Water Canker* (Wasserkrebs), or, as we should rather term it, *gangrene of the mouth*, is characterized by the appearance of a gangrenous spot in some part of the mouth, which rapidly spreads and destroys the structure of the surrounding tissues. General languor, peevishness, disinclination for play or exertion, frequent whining, irritability, tendency to sleep, but without rest; loss of appetite, increase of thirst, nausea or actual vomiting, diarrhœa or constipation, &c. are observed to precede the actual formation of the disease. Dr. Richter describes three varieties, which he terms the *scorbutic*, the *gastric*, and the *metastatic* Wasserkrebs.

*a.* In the first of these the gums become tumid, livid, hot, and bleed readily; the salivary glands swell, the secretion of saliva is increased and vitiated; the gums part from the alveolar processes; the teeth become loose and incrusted with a foul mucous secretion. After this scorbutic state has continued for a few days or weeks, grey, lead-coloured, gangrenous spots form on the gum, which rapidly increase, run one into another, and pass into a sphacelating ulcer, or become at once black and dry, and exhibit only a mass of slough. If the progress of the affection cannot be checked, the gangrene spreads to the lips and surrounding parts, even as far as the cheeks, which become swollen, hard, flushed, glistening, and burning hot. The odour becomes cadaverous, and with the appearance of low fever, accompanied with colliquative symptoms and collapse, the destructive process extends over the walls of the mouth in such a manner, that in some cases the tongue and throat may be seen from without covered with gangrenous spots. The maxillary bones themselves are attacked, and considerable portions of them, as well as the teeth, are destroyed. In such circumstances death is commonly the inevitable result, since the body, already much weakened by the existing scorbutic affection, is deprived of its remaining powers by the overwhelming force of the disease.

*b.* In the second or gastric form of the disease, the gangrene does not show itself, as in the preceding variety, first upon the gums; but after the occurrence of symptoms of disorder of the digestive system with which aphthæ are associated, it breaks out upon the inner surface of the cheek, or at one of the angles of the mouth. Increased secretion of saliva, which has a peculiar odour, with asthenic swelling, as above described, and secondary fever, shows itself, and at the same time on some part of the inner surface of the cheek, where externally a hard knot may be felt, a small discoloured vesicle appears. This vesicle, which, in consequence of the friends not apprehending change, is liable to be overlooked, bursts, and in a short time passes into a round, foul, and sphacelating ulcer, with red circumscribing edges, which continually extends itself both superficially and in depth, and at length perforates the cheek; an ash-coloured livid spot, red at the edge, having previously appeared on the external surface. The progress of the gangrene lays open the cavity of the mouth in a few days, to a great extent, attacking more or less the gums and maxilla, and the child quickly perishes with the constitutional symptoms before mentioned.

*c.* In the third form, which this author would seem to regard as the result of metastasis, though as it appears to us without sufficient grounds, the premonitory symptoms are of course the symptoms of the disease which precedes this attack, whether small-pox, scarlet fever, roseola, measles, fevers of different types, or inflammation. The destructive process in this form breaks out suddenly, and commences also in the neighbourhood of the cavity of the mouth. After the formation of an asthenic, hard tumour, a red spot arises on the mucous membrane, where externally a hard lump may be felt, which becomes gangrenous, and is circumscribed with a red border. This spot steadily increases, the mortification extending also in depth, and the progress of the destruction may be traced from hour to hour on the outer surface of the cheek, by the progress of the boundary line, as the inner surface becomes more and more involved in the disease. The formation of a sphacelating ulcer with secretion, as in the preceding variety, is not observed to occur, the destructive process resembling rather a mummification from the drying up of the dead macerated parts.

The three states here described will be readily recognized by our readers, as, though not clearly pointed out in systematic works, they are not, at least the first and third forms, of very infrequent occurrence. We cannot but think, however, that the first variety is incorrectly united with the other two, since it differs essentially both in its origin and progress. It is, in fact, nothing more than a high degree of scorbutus, and so far from being confined to children may, in its essential symptoms at least, occur in those of more advanced age. We have had occasion to observe the occurrence of the third form also in a young woman, during the course of typhoid fever, in which case the symptoms in no respect differed from those occurring in children. The patient recovered after having suffered the loss of a considerable portion of the right cheek and corresponding side of the under lip. Dr. Richter's term of

Kinderbrand, therefore, is no more appropriate than some of the names assigned to the disease which he criticises. The amount of destruction, even in some of the cases which recover, is frightful; in one which we have recently seen in a boy, eight years of age, also after fever, upwards of half the under lip, and a third of the upper lip are gone; the gums, teeth, and cavity of the mouth are exposed, and a portion of the gum and alveolar process of the under jaw destroyed. The process of reparation has, however, commenced, and there is every prospect of ultimate recovery.

2. *Mortification of the external parts of Generation in Female Infants and Children.* This affection has been noticed by several authors, British as well as foreign; among others, by J. V. Müller, Daniel, Kinder Wood, Thomson, Isnard, Cevoule, Hall, James, Burns, Wiegand, &c. It is, however, by no means of so frequent occurrence as gangrene of the mouth; and it may, perhaps, admit of a question, whether precisely the same affection is intended by the several authors enumerated. Müller, Isnard, Cevoule, and Burns, with the author of this treatise, regard this disease as identical with the gangrene of the mouth, but the cases related by Mr. Kinder Wood, (*Medico-Chirurgical Transactions*, vol. vii.) and referred to by Professor Burns, do not appear to us absolutely of the same nature with the disease described by Dr. Richter, from whom we condense the following account. After the system has been debilitated by preceding disease, there is observed general languor, headach, sickness, loss of appetite, and in very delicate children even symptoms of mild fever, which are succeeded by burning pain in the organs of generation, and the formation of a pale red, circumscribed spot on the inner surface of the labia, and on the nymphæ, together with hard swelling of the surrounding parts extending to the pubis. This state gives rise to considerable irritation and pain in passing water, and in two or three days the inner surface of the labia majora, and parts around, appear grey and ash-coloured. The spots have a circumscribed appearance, and are red at the edge, and the whole of the external parts are pale-red, hot, and swollen. If the progress of the disease is not checked, the grey colour of the spots becomes black and the mortification spreads over the perineum on the one side, and the superior commissure of the genital organs on the other. The excretion of urine becomes difficult or wholly suppressed, the pulse accelerated and small, the countenance sunken, diarrhœa supervenes, and the children die suddenly after the exhaustion has reached its highest pitch, often without any disturbance of the sensorial functions. Sometimes there is a secretion of a foul, corroding, fetid ichor; sometimes the gangrenous eschar becomes hard, and is torn off by the patients piecemeal. When, however, it is possible to put a stop to the destructive process, a line of demarcation forms at the red margin, the inflammation of the surrounding parts increases, the eschar contracts and is thrown off amid the secretion of pus, which becomes gradually of a good quality, and the ultimate loss of substance, partly from the filling up by granulation, partly from the drawing together of the neighbouring parts, is often, apparently, very inconsiderable.

This account of the symptoms and progress of the affection in the genital organs manifests, it must be owned, considerable resemblance to the gangrene of the mouth, before described; and the opinion which advocates the identity of the two affections is materially strengthened by their simultaneous occurrence in the same individual. An anonymous author, in Hecker's Annalen for April, 1829, mentions a case in a girl three years of age, in which, on the nineteenth day after the development of the disease in the mouth, gangrenous spots also appeared on the genitals, and another in which the gangrene was observed first on the genitals, and subsequently attacked the face. Notwithstanding these facts, however, a careful consideration of the subject leads us to conclude, either that the disease of the mouth and that of the organs of generation, are distinct affections, or that there exists another form of gangrene attacking the genitals of young females, in addition to the one described by Dr. Richter, and the mortification occasionally following infantile erysipelas. To this third form of gangrene must be referred the two cases related by Mr. Kinder Wood, which, whether distinct from the affection described by Dr. Richter or not, are, as it appears to us, altogether a different disease from the cases of gangrene of the mouth, which we have ourselves witnessed. The following is the first and mildest case reported by Mr. Wood, selected for quotation on account of its being the shortest.

"Miss R., aged six years, had complained three or four days of headach; had been chilly, and occasionally hot; she had been sickly, and taken little food; was dull, heavy, and languid. This morning (Jan. 22, 1815), she had complained of pain in making water: upon examination the pudendum was found inflamed; upon which I was called in. The inner surface of the left labium was ulcerated, as well as the clitoris; the right labium was inflamed, and the whole parts tumefied, of a dark purple hue, not unlike some kinds of erysipelas; the mons veneris was enlarged and inflamed; the perineum was inflamed and covered with aphthæ, which also encircled the anus; the discharge was thin, copious, and offensive, and had inflamed the top of the thigh, where it had been suffered to remain. The face had a peculiar paleness; the bowels were slow; the pulse quick and weak; the appetite diminished; the tongue of a dull clay colour, she was thirsty, complained of chilliness, and was indisposed for motion. The liquor plumbi acetatis dilutus was ordered as a lotion, to be applied lukewarm; and poultices made up with the same fluid were directed. A decoction of bark was also given with confectio cardiaca. By the use of these means, the enlargement of the parts gradually subsided, the foul bottom of the sores became red, after which the ointment of white lead was used, and the parts healed by the 14th of February."

The second case is similar but more severe, mention being again made of the occurrence of aphthæ and clusters of vesicles. What the fatal cases may have been does not appear, as they are not related.

3. *Gangrene of the Skin.* Of this form of the disease Dr. Richter relates the case of a child, in whom, after an attack of measles, which had advanced irregularly, there appeared upon the lips, near the angles of the mouth, and upon the outer surface of the cheek, several gangrenous, brownish-black spots, with red edges, from the size of a lentil to that of a fourpenny piece (Groschen), without disturbance of the general health. The gangrenous destruction had here arisen from a red spot in the outer skin, which, within forty-eight hours had acquired, first a grey, and then a black colour. The gangrenous eschars fell off and the loss of substance was soon repaired, amid the secretion of well-formed pus. In another place, near the angle of the mouth, on the right side, after the separation of the eschar, the ulcer beneath was found to be funnel-shaped, and, as was ascertained by passing a probe, had perforated the cheek. Some cases are then mentioned of gangrene occurring in the fœtal state, which, as it appears to us, have nothing whatever in common with the diseases under consideration. The following instance related by Romberg in Rust's Magazin, is more to the purpose. In a child, fifteen months old, eleven days after the eruption of the measles, a dark-brown roundish spot, of the size of a pea, appeared upon the under lip; and a second, of similar character, showed itself in the centre of the right cheek, which, in forty-eight hours, increased much in size. At the same time a round bladder, of a dark-blue colour, was observed in the interstice between the thumb and fore-finger of the left hand. On the fifth day the spots on the face had the appearance of an eschar formed by means of caustic potash, became detached, and left a perforation both of the cheek and of the lip. The bladder on the hand dried up to a black crust, which was partially loosened, and showed an apparently deep, penetrating destruction of the cellular tissue. Healthy granulations were subsequently formed, both upon the cheek and the hand. We are disposed to view both this case, and the one before mentioned, as instances of the genuine form of gangrene of the mouth; the following is of a more questionable nature, and is that to which the name of infantile gangrene of the skin more correctly applies. It is related at length by Martini, in the twenty-seventh volume of Rust's Magazine, under the name of gangræna infantilis, and occurred in a small weakly infant eight days old, and of premature birth, which was too feeble to draw milk from the breast. The nurse observed, on a sudden, black spots on both legs near the feet; when the physician was called in, he saw both legs above the joint, gangrened all round for the breadth of two inches. The skin was bluish-black, dry, cold, separated from the healthy skin of the neighbouring parts (which were not swollen) by a sharp edge. On the left foot the appearance extended over the heel and a part of the sole of the foot. There was no other appearance of disease. The surrounding skin soon inflamed, and in three days both extremities had become swollen and painful; the strength failed, a bloody swelling on the head became at the same time gangrenous, and four days from the occurrence of the first trace of disease, the child died amid convulsions. According to Billard, who describes this affection under the name of gangræna neonatorum, it is distinct from infantile erysipelas, and is owing rather to impeded circulation in the capillary vessels, and congestion of imperfectly arterialized blood in the skin and subcutaneous cellular tissue. He states, that it usually commences on the fingers and toes, though it also occurs on the arms and legs. The skin around the nails becomes bluish-red, swells or retracts; shrivels up, or becomes covered with small bladders containing a bluish serosity, which exudes and is followed by discoloured excoriations, emphysema, and a gangrenous odour. The sick remain almost motionless, insensible, scarcely respiring, and utter a hoarse plaintive cry; the abdomen swells, different parts of the body become œdematous, and gangrenous spots or petechiæ appear on the limbs and trunk.

Dr. Richter, in the treatise before us, considers next in succession the nature of the disease, the etiology, the prognosis, and the treatment.

Upon these we need not dilate at any length. The nature of these several forms of disease is sufficiently evident from what has gone before, and the reader will be at no loss to gather our own opinion as to the distinct character of some of the affections here described from the remarks which we have had occasion to make. The causes, as far as they are known, would seem to be, speaking in general terms, whatever tends to depress and debilitate the powers of the constitution, e. g. original want of power or strength in the organization, as shown in weakly children, or those prematurely born; imperfect nourishment, want of cleanliness and care, confined or impure atmosphere, and the weakening process of preceding diseases, more especially of such as are in themselves diseases of debility, or in which an asthenic state is liable to occur. The prognosis is unfavorable in proportion as these causes are in greater activity. The treatment, it is scarcely necessary to say, should consist in paying every attention to cleanliness and ventilation, and to the affording of nourishment fitted for the support of the powers of life; in the exhibition of wine, quinine, and other tonic remedies, and in the local use of lotions and poultices with chloride of lime, camphor, and other means, which the greater or less tendency to sloughing on the one hand, or to inflammation on the other, will readily suggest to the well-informed and observant practitioner.

We may, in conclusion, state that we are inclined to recognize the following distinct forms of infantile gangrene, as described by Dr. Richter and others: 1st. Gangrene of the gums and mouth arising from a high degree of scorbutus; this is Dr. Richter's first variety of cancer aquaticus. 2d. Gangrene of the mouth from loss of power, including the second and third varieties of cancer aquaticus, with some of the cases referred by the author to gangrene of the skin. 3d. Sphacelus of the genitals, nearly allied to the preceding form, and sometimes occurring in connexion with it. 4th. Vesicular inflammation (herpes?) of the labia, with tendency to gangrene, of which the cases related by Mr. Wood are examples. 5th. Infantile gangrene of the skin, described by Billard, under the name of gangræna neonatorum, to which a part of Dr. Richter's description applies. 6th. The gangrene which occasionally follows erysipelas in infants.

---

